# A percepção da audição está relacionada à ocorrência de quedas entre idosos durante a pandemia de COVID-19? Uma análise longitudinal

**DOI:** 10.1590/0102-311XPT022824

**Published:** 2024-11-22

**Authors:** Vitória Neves de Barros, Thamara Hubler Figueiró, Danúbia Hillesheim, Eleonora d’Orsi

**Affiliations:** 1 Universidade Federal de Santa Catarina, Florianópolis, Brasil.

**Keywords:** Audição, Perda Auditiva, Idoso, Acidentes por Quedas, COVID-19, Hearing, Hearing Loss, Aged, Accidental Falls, COVID-19, Audición, Pérdida Auditiva, Anciano, Accidentes por Caídas, COVID-19

## Abstract

O objetivo deste estudo foi estimar a associação entre a percepção da audição ao longo do tempo e a ocorrência de quedas entre idosos durante a pandemia de COVID-19. Tratou-se de um estudo longitudinal, com dados da terceira onda de entrevistas domiciliares (2017/2019) e da quarta onda de entrevistas por telefone do estudo EpiFloripa Idoso (2021/2022), uma coorte de base populacional com idosos de 60 anos ou mais, realizada desde 2009 na cidade de Florianópolis, Santa Catarina, Brasil. A ocorrência de queda no último ano foi definida como a variável dependente neste estudo, enquanto a percepção da audição ao longo do tempo foi a variável independente. Foi realizada análise de regressão logística para identificar a relação entre a percepção da perda auditiva entre a terceira e a quarta onda do estudo com a ocorrência de quedas. Participaram do estudo 289 idosos, sendo a maioria do sexo feminino (69,1%), na faixa etária de 70 a 79 anos (53,4%) e com 12 anos ou mais de escolaridade (41%). Pessoas idosas que se mantiveram com dificuldade auditiva apresentaram 181% mais chance (OR = 2,81; IC95%: 1,08-7,34) de sofrer queda, quando comparado com as pessoas sem dificuldade auditiva. Em conclusão, os resultados deste estudo fornecem evidências da associação entre dificuldade auditiva em pessoas idosas e maior chance de quedas. Esses resultados sugerem a necessidade de intervenções que visem a reabilitação auditiva. Ainda, uma abordagem integrada e multifacetada é fundamental para mitigar os riscos de quedas nesse grupo etário, considerando tanto as necessidades auditivas quanto as medidas de prevenção de quedas.

## Abstract (en)

This study estimated the association between hearing perception over time and the occurrence of falls among older adults during the COVID-19 pandemic. This is a longitudinal study, with data from the third wave of household interviews (2017/2019) and the fourth wave of telephone interviews from the EpiFloripa Idoso study (2021/2022), a population-based cohort with older adults aged 60 years or older, carried out since 2009 in the city of Florianópolis, state of Santa Catarina, Brazil. Fall event in the last year was the dependent variable in this study, while the perception of hearing over time was the independent variable. Logistic regression analysis was performed to identify the relation of the perception of hearing loss between the third and fourth waves of the study and the occurrence of falls. A total of 289 older adults participated in the study, mostly females (69.1%), aged 70 to 79 years (53.4%), and with 12 or more years of schooling (41%). Older adults who remained with impaired hearing were 181% more likely (OR = 2.81; 95%CI: 1.08-7.34) of falling, when compared to those without impaired hearing. In conclusion, this study results provide evidence of the association between hearing difficulty in older people and a higher chance of falls. Outcomes suggest the need for interventions aimed at auditory rehabilitation. Furthermore, an integrated and multifaceted approach is essential to mitigate the risks of falls in this age group, considering both hearing needs and fall prevention measures.

## Resumen

El objetivo de este estudio fue estimar la asociación entre la percepción auditiva a lo largo del tiempo y la incidencia de caídas entre ancianos durante la pandemia de COVID-19. Se trata de un estudio longitudinal, con datos de la tercera ola de entrevistas en hogares (2017/2019) y de la cuarta ola de entrevistas telefónicas del estudio EpiFloripa Anciano (2021/2022), una cohorte de base poblacional con personas de 60 años o más, realizado desde el 2009 en la ciudad de Florianópolis, Santa Catarina, Brasil. La incidencia de caídas en el último año se definió como la variable dependiente en este estudio, mientras que la percepción auditiva a lo largo del tiempo fue la variable independiente. Se realizó un análisis de regresión logística para identificar la relación entre la percepción de la pérdida auditiva entre la tercera y la cuarta ola del estudio y la incidencia de caídas. Participaron en el estudio 289 ancianos, la mayoría del sexo femenino (69,1%), con edades entre 70 y 79 años (53,4%) y con 12 años o más de escolaridad (41%). Los ancianos que permanecieron con dificultades auditivas tuvieron 181% más probabilidades (OR = 2,81; IC95%: 1,08-7,34) de sufrir una caída, en comparación con las personas que permanecieron sin dificultades auditivas. En conclusión, los resultados de este estudio aportan evidencias de la asociación entre la dificultad auditiva en ancianos y una mayor probabilidad de sufrir una caída. Estos resultados sugieren la necesidad de intervenciones dirigidas a la rehabilitación auditiva. Además, es esencial un enfoque integrado y multifacético para mitigar los riesgos de caídas en este rango de edad, teniendo en cuenta tanto las necesidades auditivas como las medidas para prevenir las caídas.

## Introdução

A queda em idosos é um evento multifatorial, que envolve alterações fisiológicas, tanto decorrentes do envelhecimento natural quanto causadas pelo uso de medicamentos, juntamente com fatores comportamentais que dizem respeito à forma como os idosos interagem com o ambiente ^1^. Em linhas gerais, quedas são definidas por uma mudança de posição inesperada, não intencional ou dependente de força externa ^2^. Elas representam uma das principais origens de lesões não intencionais e, com frequência, resultam em busca por atendimento médico de emergência ^3^. Com isso, é possível que o idoso desenvolva complicações físicas e mentais, o que pode levar à diminuição de sua funcionalidade ou, em casos extremos, resultar em óbito ^3^.

Entende-se que a ocorrência de quedas em idosos representa uma questão de saúde pública, uma vez que cerca de 28% a 35% dos idosos sofrem algum episódio a cada ano, porcentagem que aumenta após os 70 anos de idade, chegando a 42% ^4^. De 2000 a 2019, foram registrados 135.209 óbitos relacionados a quedas de idosos no Brasil, observando-se uma tendência crescente nas taxas de mortalidade por esse agravo no período, notavelmente maior em pessoas de 80 anos ou mais ^5^.

Assim como a incidência de quedas, a perda auditiva é uma condição frequente na população idosa ^6^, podendo ser causada por infecções, perdas induzidas por ruído, complicações relacionadas a doenças e, principalmente, em decorrência do envelhecimento. Segundo a Organização Mundial da Saúde (OMS), uma pessoa possui perda auditiva se sua capacidade auditiva está reduzida em comparação com os padrões de normalidade, que correspondem a limiares menores que 20 decibéis ^7^. A presbiacusia, perda auditiva relacionada ao envelhecimento, é comum entre os idosos e está atrelada a consequências importantes, como fragilidade física, déficits cognitivos, isolamento social e depressão ^8^. Essa condição tem uma origem multifatorial, resultante de uma combinação de fatores, como idade, sexo, comportamentos (incluindo tabagismo e alcoolismo) ^8^, exposição a agentes ototóxicos e ruídos intensos ^9^ ^,^ ^10^.

Quedas e deficiências auditivas em idosos, individualmente, são fatores relevantes que impactam a funcionalidade, a autonomia e a saúde dessa população ^11^. Estudos indicam que a presença de deficiência auditiva representa um fator de risco significativo para quedas ^12^ ^,^ ^13^. O mecanismo potencial subjacente a essa relação pode envolver o comprometimento da função vestibular relacionada à navegação espacial e à memória, resultante da deficiência auditiva. Isso pode levar a uma redução no equilíbrio, aumentando, consequentemente, o risco de quedas ^13^. Além disso, a literatura sugere uma relação direta entre a gravidade da perda auditiva e o aumento do risco de quedas ^12^. No entanto, é importante destacar que há diferentes perspectivas na literatura. Segundo Heitz et al. ^11^, embora a perda auditiva seja considerada um marcador clínico para o risco de quedas, não é necessariamente um fator de risco independente para quedas não-fatais.

Em estudo de Zayat et al. ^14^, foi constatado que, durante a pandemia de COVID-19, a incidência de quedas entre idosos se manteve similar em relação ao período anterior à pandemia. Outro estudo, conduzido por Nguyen et al. ^15^, investigou a prevalência de quedas em idosos no Vietnã durante a pandemia, revelando uma taxa de 23,1% de quedas, das quais 24,5% ocorreram de forma recorrente (duas ou mais quedas nos últimos 12 meses). No entanto, em relação à ocorrência de quedas associadas à perda auditiva durante o período pandêmico, há carência de estudos que se concentrem nesse tópico específico. Entre os poucos autores que o abordaram, há discussão sobre a possibilidade de que a pandemia de COVID-19 possa aumentar os eventos de queda devido à redução da atividade física em idosos, o que poderia resultar em maior fragilidade ^15^.

Compreender a relação entre a percepção auditiva ao longo do tempo e a ocorrência de quedas em idosos do sul do Brasil, no período pandêmico, não apenas enriquecerá o conhecimento científico acerca dos impactos da pandemia, mas também terá o potencial de reforçar as políticas de prevenção de quedas e a promoção da saúde auditiva. Além disso, o estudo poderá fornecer percepções valiosas para profissionais da saúde, formuladores de políticas e cuidadores, desempenhando papel fundamental na identificação de grupos suscetíveis e na aplicação de abordagens específicas para atenuar possíveis efeitos adversos da pandemia na saúde e no bem-estar dos idosos.

Diante desse contexto, o objetivo deste estudo foi estimar a associação entre a percepção da audição ao longo do tempo e a ocorrência de quedas entre idosos durante a pandemia de COVID-19.

## Métodos

### Delineamento e local do estudo

Trata-se de um estudo longitudinal realizado com dados das ondas 3 e 4 do estudo de coorte EpiFloripa Idoso (https://epifloripaidoso.paginas.ufsc.br/). O estudo é uma coorte de base populacional e domiciliar realizada com idosos de 60 anos ou mais que vivem em áreas urbanas do Município de Florianópolis, Santa Catarina, Brasil. Os dados utilizados foram coletados em duas ondas distintas: a terceira onda, que consistiu em entrevistas domiciliares realizadas entre 2017 e 2019 com 1.335 idosos; e a quarta onda, que envolveu uma subamostra de entrevistas por telefone entre os anos de 2021 e 2022 com 363 idosos.

Informações detalhadas sobre o plano amostral, os aspectos operacionais das entrevistas domiciliares e as estratégias empregadas na terceira onda da pesquisa (2017/2019) foram descritas previamente ^16^. Na onda 4 do estudo EpiCovid Tel, foram incluídas todas as pessoas entrevistadas na onda 3, tanto aquelas do acompanhamento quanto aquelas consideradas como perda ou recusa na onda 3 (das perdas e recusas da onda 3 foram entrevistadas 20 pessoas na onda 4). Dessa forma, foi realizada tentativa de entrevista por telefone, entre novembro de 2021 e novembro de 2022, com todos os elegíveis na terceira onda do acompanhamento da coorte, incluindo entrevistados, recusas e perdas. Entre os critérios de exclusão do EpiCovid Tel estavam: (1) perdas e recusas dos participantes do EpiFloripa Adulto que foram convidados a fazer parte da pesquisa na onda 3; (3) novos participantes selecionados que se recusaram a fornecer entrevistas domiciliares na onda 3, e portanto, sem informações para o estudo.

Os entrevistadores da onda EpiCovid Tel foram alunos de graduação e pós-graduação, os quais foram treinados previamente sobre a abordagem ao telefone e o preenchimento do questionário eletrônico. Os dados dessa onda do estudo foram coletados e gerenciados usando a ferramenta eletrônica de captura de dados denominada Research Electronic Data Capture (REDCap; https://redcapbrasil.com.br/), hospedada nos servidores da Universidade Federal de Santa Catarina ^17^ ^,^ ^18^, a qual é uma plataforma de software segura baseada na *web* e projetada para apoiar a captura de dados para estudos de pesquisa. Os entrevistadores contavam, ainda, com um manual sobre os conhecimentos relativos à pesquisa e com documentos contendo o fluxo das ligações. Além disso, os participantes que não puderam responder ao questionário por telefone (em virtude de algum comprometimento cognitivo ou auditivo) tiveram a possibilidade de permitir que um informante respondesse à entrevista para seções específicas.

### Perdas, recusas e óbitos

Os óbitos foram detectados durante as chamadas telefônicas, que incluíam interação com parentes e amigos dos participantes. Quando um familiar ou amigo informava sobre um óbito, eram requisitadas informações sobre a data, a causa e o local do falecimento para registro na base de dados. Por outro lado, as recusas foram manifestadas verbalmente durante as chamadas, enquanto as perdas foram determinadas após no mínimo três tentativas de contato em dias e períodos diferentes, incluindo fins de semana.

### Variável dependente

A variável dependente deste estudo foi a ocorrência de queda no último ano, avaliada por meio da pergunta: “O(a) Sr(a) sofreu alguma queda no último ano?”, com opção de resposta “Não” ou “Sim”. Para elucidar a definição de queda aos entrevistados, foi enfatizada a seguinte frase: “Eu gostaria de esclarecer para o(a) Sr.(a) que uma queda é qualquer tombo que o(a) Sr.(a) tenha sofrido, mesmo que isso NÃO tenha causado nenhum tipo de ferimento ou outro problema qualquer”.

### Variável independente

A percepção da audição foi utilizada como variável de exposição principal neste estudo, avaliada ao longo do tempo. A percepção de perda auditiva foi verificada nas duas ondas pela pergunta: “O(a) Sr(a) sente que tem dificuldade para ouvir?” cuja resposta poderia ser “Não” ou “Sim”. Foi identificada a possível mudança da percepção auditiva na onda 3 para a onda 4, sendo categorizada como: “manteve sem dificuldade auditiva”, “passou a ter dificuldade auditiva”, “deixou de ter dificuldade auditiva” e “manteve com dificuldade auditiva”. Embora o autorrelato tenha limitações devido à sua natureza subjetiva, pesquisadores indicam que a percepção auditiva em pesquisas populacionais apresenta valores satisfatórios de sensibilidade e especificidade ^19^.

### Covariáveis

Com base em literatura prévia ^11^ ^,^ ^12^ ^,^ ^13^ foram considerados como fatores de confusão as características dos participantes na linha de base do estudo (onda 3): sexo (masculino; feminino); faixa etária (60 a 69; 70 a 79; 80 anos ou mais); escolaridade (0 a 8 anos; 9 a 11 anos; 12 anos ou mais); e uso de aparelho auditivo (não; sim). Ainda, incluiu-se o tabagismo, avaliado pela pergunta “O(a) Sr(a) fuma ou fumou cigarros?” (nunca; fumou e parou; fuma atualmente) e o consumo indevido de álcool, investigado pelo *Alcohol Use Disorders Identification Test-Concise* (AUDIT-C; Teste de Identificação de Transtornos por Uso de Álcool - Conciso) ^20^, contendo três perguntas que totalizam 12 pontos, com pontuações de seis ou mais indicando alto risco, definido neste estudo como consumo indevido. Já a prática de atividade física moderada a vigorosa no lazer foi considerada quando o participante referiu realizá-la por pelo menos 150 minutos na semana ^21^.

Também foi avaliado o comprometimento cognitivo por meio do *Mini-exame do Estado Mental* ^22^, sendo que os idosos sem escolaridade e com pontuação ≤ 19 no teste ou aqueles com alguma escolaridade e pontuação ≤ 23 foram considerados com a presença de comprometimento cognitivo ^23^. A presença de algum grau de incapacidade nas atividades de vida diárias foi avaliada por meio do *Questionário Brasileiro de Avaliação Funcional Multidimensional*, adaptado do questionário *Old Americans Resources and Services* (BOMFAQ/OARS) ^24^, categorizada como “nenhuma atividade de vida diária”, “uma a três atividades de vida diárias” e “quatro ou mais atividades de vida diárias” ^25^. A presença de diabetes mellitus (não; sim), hipertensão arterial sistêmica (não; sim), acidente vascular cerebral (não; sim) e doença cardiovascular (não; sim) foram investigadas mediante a pergunta: “Algum médico ou profissional de saúde já disse que o(a) Sr(a) tem/teve:”. O índice de massa corporal (IMC) foi calculado pelo peso em quilogramas dividido pela altura ao quadrado, sendo que ambas as medidas foram avaliadas nas entrevistas domiciliares da onda 3 mediante técnica específica.

### Análise dos dados

A normalidade da variável IMC foi testada por meio do teste de Shapiro-Wilk e, graficamente, por meio de histogramas. Para a descrição das variáveis qualitativas, os dados foram representados por meio de frequências absolutas e relativas, com seus respectivos intervalos de 95% de confiança (IC95%), enquanto as variáveis quantitativas foram apresentadas por meio da média.

Para verificar a diferença de proporções entre as variáveis de características da amostra e em relação ao desfecho, utilizou-se o teste qui-quadrado de Pearson e o teste de Kruskal-Wallis. Quando os pressupostos do teste qui-quadrado não foram atendidos, utilizou-se o teste exato de Fisher. Para verificar a diferença entre medidas de tendência central entre a variável IMC e o desfecho (ocorrência de quedas) foi empregado o teste t de Student e, quando comparado entre o *status* da onda 4, foi utilizado o teste de Kruskal-Wallis.

Tanto para a análise bruta quanto para a ajustada, a *odds ratio* (OR) foi utilizada como medida de associação, estimada por meio da análise de regressão logística. A análise de regressão foi conduzida exclusivamente com os dados da amostra que apresentavam preenchimento completo para todas as variáveis de interesse (amostra analítica). A variável de exposição principal foi ajustada por todas as covariáveis do estudo, independentemente do valor de p, por meio de método “*enter*”. O nível de significância adotado foi de 5%. A análise dos dados foi conduzida no software Stata, versão 14.0 (https://www.stata.com), considerando-se o delineamento do estudo e o peso amostral no banco de dados (comando *svy*). A análise pós-estimação foi conduzida para verificar o resultado do teste de Hosmer-Lemeshow, que indicou o ajuste adequado do modelo.

### Aspectos éticos

O estudo EpiFloripa 2017/2019 recebeu aprovação como uma emenda do EpiFloripa 2013/2014 pelo Comitê de Ética em Pesquisa com Seres Humanos da Universidade Federal de Santa Catarina (CAAE: 16731313.0.0000.0121). A quarta onda do estudo também obteve aprovação pela mesma instituição (CAAE: 48422821.5.0000.0121). Além disso, todos os idosos que concordaram em participar da pesquisa assinaram o Termo de Consentimento Livre e Esclarecido (TCLE). Para as entrevistas por telefone, o consentimento foi obtido verbalmente.

## Resultados

A Tabela 1 tem o propósito de caracterizar a amostra total da onda 3 (linha de base neste estudo) e investigar essas características segundo o *status* de acompanhamento (entrevistados, recusas, perdas e óbitos) da onda 4. Na onda 3, houve maior percentual de mulheres (63%), pessoas entre 70 a 79 anos (43,7%) e com até 8 anos de estudo (50,3%). Com relação à percepção auditiva, 30,2% dos indivíduos relataram ter dificuldade para ouvir na onda 3, e 31,8% sofreram alguma queda nessa mesma onda (Tabela 1).

**Tabela 1:** Descrição dos participantes da onda 3 (linha de base) e de acordo com o *status* de acompanhamento na onda 4 (EpiCovid Tel). Estudo EpiFloripa Idoso 2017/2019, Florianópolis, Santa Catarina, Brasil, 2022.

Variáveis	Onda 3 (2017/2019) [n = 1.335]		Onda 4 (EpiCovid Tel - 2021/2022)								Valor de p *
			Entrevistados [n = 343 (25,7%)]		Recusas [n = 220 (16,5%)]		Perdas [n = 706 (52,9%)]		Óbitos [n = 66 (4,9%)]		
	n	%	n	%	n	%	n	%	n	%	
Sexo											0,050
Masculino	521	37,0	121	20,2	98	19,3	267	53,9	35	6,6	
Feminino	814	63,0	222	28,4	122	14,8	439	52,0	31	4,8	
Faixa etária (anos)											< 0,001
60-69	461	30,2	128	28,5	80	18,8	244	51,0	9	1,7	
70-79	554	43,7	153	28,3	98	17,3	283	50,4	20	4,0	
80 ou mais	320	26,1	62	16,7	42	12,4	179	58,6	37	12,3	
Escolaridade (anos)											0,221
0-8	671	50,3	141	21,5	115	17,3	377	56,0	38	5,2	
9-11	228	16,2	60	26,4	48	19,7	111	48,8	9	5,1	
12 ou mais	431	33,5	141	30,6	56	13,3	215	49,9	19	6,2	
Tabagismo											0,225
Nunca	784	59,8	205	25,6	127	16,1	414	52,4	38	5,9	
Fumou e parou	429	30,9	111	25,1	79	19,3	214	49,6	25	6,0	
Fuma atualmente	122	9,3	27	24,3	14	8,9	78	65,4	3	1,4	
Consumo de álcool indevido											0,139
Não	1.062	81,1	259	24,4	174	16,2	570	53,2	59	6,2	
Sim	271	18,9	84	29,5	45	17,6	135	50,7	7	2,2	
Prática de atividade física moderada a vigorosa											0,029
Não	1.155	88,0	283	24,2	186	16,0	629	53,9	57	5,9	
Sim	160	12,0	57	36,0	32	21,0	65	40,9	6	2,1	
Uso de aparelho auditivo											0,527 **
Não	1.240	92,4	321	25,8	202	16,3	658	52,3	59	5,6	
Sim	95	7,6	22	18,9	18	18,6	48	58,4	7	4,1	
Comprometimento cognitivo											0,018
Não	1.065	79,5	293	27,3	187	17,2	544	50,9	41	4,6	
Sim	258	20,5	48	18,0	32	13,6	153	59,2	25	9,2	
Dependência nas atividades de vida diárias											0,037
Nenhuma	403	29,2	124	30,6	77	18,9	194	48,2	8	2,3	
1-3	471	36,4	118	25,5	83	17,5	247	50,9	23	6,1	
4 ou mais	436	34,4	92	20,8	56	13,0	255	59,0	33	7,2	
Hipertensão											0,350
Não	516	40,5	142	26,5	82	16,6	269	53,0	23	3,9	
Sim	819	59,5	201	24,5	138	16,3	437	52,5	43	6,7	
Diabetes											0,946
Não	999	74,3	260	25,6	162	16,2	529	52,9	48	5,3	
Sim	336	25,7	83	24,6	58	17,1	177	52,2	18	6,1	
Acidente vascular cerebral											0,026
Não	1.195	90,4	313	26,1	204	17,1	626	51,8	52	5,0	
Sim	140	9,6	30	18,4	16	10,3	80	61,1	14	10,2	
Doenças cardiovasculares											0,044
Não	932	69,8	246	27,5	165	17,4	486	50,4	35	4,7	
Sim	403	30,2	97	20,2	55	14,2	220	58,2	31	7,4	
Percepção auditiva											0,294
Não tem dificuldade para ouvir	908	69,8	233	26,4	145	14,9	488	53,6	42	5,1	
Tem dificuldade para ouvir	374	30,2	102	24,6	73	21,3	184	48,4	15	5,7	
Queda											0,236
Não	934	68,2	250	26,1	170	18,1	473	51,3	41	4,5	
Sim	397	31,8	92	23,4	50	13,2	231	56,2	24	7,2	
	n	Média	n	Média	n	Média	n	Média	n	Média	Valor de p
IMC	1.257	28,0	332	28,0	214	28,1	659	28,2	52	26,4	0,268 ***

IC95%: intervalo de 95% de confiança; IMC: índice de massa corporal.

* Teste qui-quadrado de Pearson considerando o peso amostral;

** Teste exato de Fisher;

*** Teste de Kruskal-Wallis.

Quando observada a distribuição das entrevistas realizadas, recusas, perdas e óbitos na onda 4 (EpiCovid Tel), em relação às características sociodemográficas e condições de saúde da onda 3, houve diferença estatisticamente significativa em relação à faixa etária, prática de atividade física moderada a vigorosa, comprometimento cognitivo, dependência nas atividades de vida diárias, acidente vascular cerebral e doenças cardiovasculares (p < 0,05). De forma geral, houve maior percentual de mulheres entrevistadas na onda 4 (28,4% *vs.* 20,2%; p = 0,05) e de idosos na faixa etária de 60 a 69 anos (28,5%), com maior percentual de óbitos entre aqueles na faixa etária de 80 anos ou mais (12,3%). Ainda, houve maior percentual de entrevistados entre aqueles sem comprometimento cognitivo e sem dependência nas atividades de vida diárias (Tabela 1).

A Tabela 2 descreve as características da amostra analítica e a prevalência de quedas de acordo com elas (n = 289). Observou-se maior prevalência do sexo feminino (69,1%), de idosos na faixa etária de 70 a 79 anos (53,4%) e com 12 anos ou mais de estudo (41%). Além disso, mais da metade da amostra referiu nunca ter fumado (59,8%) e pouco mais de 3/4 não realizava o consumo excessivo de álcool (Tabela 2).

**Tabela 2:** Descrição das características da amostra analítica na linha de base e prevalência de quedas na onda 4 do estudo segundo estas características. Estudo EpiFloripa Idoso 2017/2019, Florianópolis, Santa Catarina, Brasil (n = 289).

Variáveis	Total		Não sofreu queda			Sofreu queda			Valor de p *
	n	%	n	%	IC95%	n	%	IC95%	
Sexo									0,006
Masculino	104	30,9	86	87,4	77,7-93,3	18	12,6	6,7-22,3	
Feminino	185	69,1	131	64,5	51,0-76,0	54	35,5	24,0-49,0	
Faixa etária (anos)									0,310
60-69	123	38,0	97	78,9	63,7-88,8	26	21,1	11,2-36,3	
70-79	139	53,4	101	66,6	56,1-75,6	38	33,4	24,4-43,9	
80 ou mais	27	8,6	19	70,3	41,6-88,8	8	29,7	11,2-58,4	
Escolaridade (anos)									0,350
0-8	108	40,7	81	73,0	57,2-84,6	27	27,0	15,4-42,8	
9-11	53	18,3	41	80,8	67,9-89,4	12	19,2	10,6-32,1	
12 ou mais	128	41,0	95	66,0	51,8-77,8	33	34,0	22,2-48,2	
Tabagismo									0,836
Nunca	172	59,8	129	73,3	62,7-81,8	43	26,7	18,2-37,3	
Fumou e parou	91	29,9	68	70,1	54,7-81,9	23	29,9	18,1-45,3	
Fuma atualmente	26	10,3	20	65,6	30,6-89,2	6	34,4	10,8-69,4	
Consumo de álcool indevido									0,969
Não	214	76,5	160	71,6	62,6-79,2	54	28,4	20,8-37,4	
Sim	75	23,5	57	71,3	52,3-84,9	18	28,7	15,1-47,7	
Prática de atividade física moderada a vigorosa									0,451
Não	241	82,9	184	70,5	61,0-78,5	57	29,5	21,5-39,0	
Sim	48	17,1	33	76,6	59,9-87,8	15	23,4	12,2-40,1	
Uso de aparelho auditivo									1,000 **
Não	280	96,4	210	72,9	65,2-79,5	70	27,1	20,5-34,8	
Sim	9	3,6	7	35,5	6,3-81,8	2	64,5	18,2-93,7	
Comprometimento cognitivo									0,673
Não	263	89,6	198	72,2	62,4-80,2	65	27,8	19,8-37,6	
Sim	26	10,4	19	66,3	37,7-86,5	4	33,7	13,5-62,3	
Dependência nas atividades de vida diárias									0,264
Nenhuma	113	36,9	92	78,7	67,6-86,8	21	21,3	13,2-32,4	
1-3	109	40,2	80	69,6	55,3-80,9	29	30,4	19,1-44,7	
4 ou mais	67	22,9	45	63,5	44,5-79,1	22	36,5	20,9-55,5	
Hipertensão									0,596
Não	124	43,9	94	69,4	57,4-79,2	30	30,6	20,8-42,6	
Sim	165	56,1	123	73,3	61,5-82,5	42	26,7	17,5-38,5	
Diabetes									0,605
Não	224	76,5	170	72,6	64,2-79,7	54	27,4	20,3-35,8	
Sim	65	23,5	47	68,2	48,2-83,1	18	31,8	16,9-51,8	
Acidente vascular cerebral									0,772 **
Não	272	96,3	205	71,5	62,3-79,2)	67	28,5	20,8-37,7	
Sim	17	3,7	12	72,8	47,2-88,9)	5	27,2	11,1-52,8	
Doenças cardiovasculares									0,955
Não	212	79,7	158	71,7	61,4-80,1	54	28,3	19,9-38,6	
Sim	77	20,3	59	71,1	50,4-85,60	18	28,9	14,4-49,6	
Percepção da audição									0,537 **
Manteve sem dificuldade auditiva	167	58,4	130	78,8	67,8-86,7	37	21,2	13,3-32,2	
Passou a ter dificuldade auditiva	44	15,5	30	67,7	51,3-80,7	14	32,3	19,3-48,7	
Deixou de ter dificuldade auditiva	16	4,2	12	78,4	51,7-92,5	4	21,6	7,5-48,3	
Manteve com dificuldade auditiva	62	21,9	45	53,8	30,3-75,7	17	46,2	24,3-69,7	
Queda na onda 4									-
Não	217	71,6	-		-	-		-	
Sim	72	28,4	-		-	-		-	
	n	Média	n	Média	IC95%	n	Média	IC95%	Valor de p
IMC	289	28,2	217	28,2	27,3-29,1	72	28,2	27,1-29,4	0,451 ***

IC95%: intervalo de 95% de confiança; IMC: índice de massa corporal.

* Teste qui-quadrado de Pearson considerando o peso amostral;

** Teste exato de Fisher;

*** Teste de Kruskal-Wallis.

Ainda, 10,4% apresentavam comprometimento cognitivo e a maioria dos participantes tinha dependência em uma a três atividades de vida diárias (40,2%). A prevalência de hipertensão arterial e diabetes na amostra analítica foi de 56,1% e 23,5%, respectivamente. Com relação à audição, apenas 3,6% da amostra usava aparelho auditivo, e se manter sem dificuldade auditiva na passagem da onda 3 para a onda 4 foi a mudança mais frequente (58,4%), além da manutenção da dificuldade auditiva (21,9%). Já a ocorrência de quedas nos últimos 12 meses, na onda 4, foi referida por 28,4% dos entrevistados (Tabela 2).

Com relação às quedas, houve maior prevalência do desfecho entre as mulheres (35,5%) quando comparado aos homens (12,6%; p = 0,006). Também foi observada maior prevalência de quedas entre aqueles com 70 a 79 anos (33,4%), que usavam aparelho auditivo (64,5%) e com comprometimento cognitivo (33,7%). A ocorrência de quedas também foi mais elevada entre aqueles que se mantiveram com dificuldade auditiva em ambas as ondas do estudo (46,2%) e os que passaram a ter dificuldade auditiva entre a onda 3 e a onda 4 (32,3%), porém sem significância estatística (p = 0,537) (Tabela 2).

A análise de associação bruta indicou que a percepção auditiva ao longo do tempo não foi associada com a ocorrência de quedas no período pandêmico (p = 0,072). Após ajuste do modelo, pessoas idosas que se mantiveram com dificuldade auditiva apresentaram 181% mais chance (OR = 2,81; IC95%: 1,08-7,34; p = 0,034) de sofrer queda, quando comparadas às pessoas que se mantiveram sem dificuldade auditiva (Tabela 3).

**Tabela 3:** Associação entre percepção da audição ao longo do tempo e ocorrência de quedas. Estudo EpiFloripa Idoso 2017/2019 - 2021/2022, Florianópolis, Santa Catarina, Brasil (n = 289).

Variável	Bruta			Ajustada *		
	OR	IC95%	Valor de p	OR	IC95%	Valor de p
Percepção da audição						
Manteve sem dificuldade auditiva	Referência			Referência		
Passou a ter dificuldade auditiva	1,77	0,72-4,31	0,209	1,85	0,76-4,54	0,175
Deixou de ter dificuldade auditiva	1,02	0,28-3,68	0,973	1,55	0,26-9,34	0,629
Manteve com dificuldade auditiva	3,18	0,94-10,79	0,063	2,81	1,08-7,34	0,034

IC95%: intervalo de 95% de confiança; OR: *odds ratio*.

Nota: ajuste do modelo avaliado pelo teste de Hosmer-Lemeshow: F(9,90) = 0,93; p = 0,506.

* Ajustado por: sexo, faixa etária, escolaridade, tabagismo, consumo de álcool indevido, prática de atividade física moderada a vigorosa, uso de aparelho auditivo, comprometimento cognitivo, dependência nas atividades de vida diárias, índice de massa corporal, hipertensão arterial, diabetes, acidente vascular cerebral e doença cardiovascular.

## Discussão

Nesta pesquisa, constatou-se que os participantes com dificuldade auditiva apresentaram 181% mais chance de sofrer queda, quando comparado com as pessoas que se mantiveram sem dificuldade auditiva. Além disso, houve maior prevalência de quedas em mulheres e entre aqueles com 70 a 79 anos. Ainda, observa-se, na amostra do EpiFloripa Idoso, que 32,3% dos envolvidos no estudo passaram a ter dificuldade auditiva autorreferida entre a onda 3 e a onda 4.

Neste estudo, cerca de 1/3 dos idosos passaram a ter dificuldade para ouvir, um fenômeno esperado com o avanço da idade ^26^. Devido aos avanços na assistência médica individual e na saúde pública, a proporção da população acima de 65 anos cresce diariamente, e, consequentemente, as condições que afetam essa população seguem aumentando. A prevalência da perda auditiva é frequente entre os idosos, afetando mais de 80% das pessoas com mais de 80 anos em algum grau ^27^. Várias mudanças ocorrem no sistema auditivo ao longo do tempo, como a degeneração neuronal e das células sensoriais, especialmente das células ciliadas externas da cóclea ^28^. No entanto, destaca-se que as interações entre a genética subjacente e as exposições ambientais são determinantes para a audição observada no final da vida ^28^.

Foi observada uma maior prevalência de quedas entre as mulheres. Esses resultados são consistentes com estudos anteriores, nos quais se destaca que a população feminina apresenta maiores índices de queda, possivelmente devido ao fato de apresentarem maiores índices de fragilidade decorrente da idade ^29^ ^,^ ^30^ ^,^ ^31^. De forma similar ao estudo de Wang et al. ^13^, houve maior prevalência de quedas entre as mulheres quando comparado com os homens.

Ainda, observou-se que a faixa etária que mais apresentou quedas neste estudo foi de 70 a 79 anos (33,4%), seguido da faixa-etária de 80 anos ou mais (29,7%). Esses resultados estão em concordância com a literatura, que aponta os idosos mais jovens (60 a 69 anos) como os menos afetados por quedas ^32^ ^,^ ^33^. Esses achados podem ser atribuídos ao declínio associado à idade nas funções físicas, sensoriais e cognitivas, somado ao aumento do número de comorbidades ^33^ ^,^ ^34^.

Os dados desta pesquisa, indicam que idosos com dificuldade auditiva apresentaram maiores chances de sofrer quedas. Ainda, aqueles que passaram a ter dificuldade auditiva também apresentaram maior probabilidade do desfecho, embora sem associação. Existem várias teorias que buscam compreender a possível associação independente entre a perda auditiva, o equilíbrio e as quedas. Dentre essas teorias, destaca-se uma possível disfunção compartilhada entre os sistemas sensoriais coclear e vestibular, assim como a necessidade de pistas auditivas para uma percepção ambiental adequada, que podem ser prejudicadas pela perda auditiva. Além disso, menciona-se que a redução na atenção em indivíduos com perda auditiva pode prejudicar a capacidade de controlar o equilíbrio em ambientes muito ruidosos ^35^. Ademais, a perda auditiva tem impactos significativos nas habilidades cognitivas dos idosos ^36^, que, por sua vez, influenciam no equilíbrio ^37^ e caminhada dos indivíduos ^35^ ^,^ ^37^.

Uma revisão sistemática ^38^ evidenciou que a amplificação auditiva em idosos melhora a orientação espaçotemporal, especialmente com o uso de implantes cocleares, e que o processo de utilização da informação auditiva para o controle do equilíbrio requer um período de adaptação. Dessa forma, ressalta-se a importância da intervenção precoce nessa população. Políticas públicas referentes à saúde auditiva demonstram-se essenciais para a manutenção da saúde da população idosa, posto que o comprometimento auditivo é comum entre ela, com implicações profundas em sua qualidade de vida ^39^. Portanto, o desenvolvimento e a implementação de políticas que abrangem a prevenção, o diagnóstico precoce e o acesso aos serviços de saúde auditiva são cruciais. Isso não apenas auxilia na diminuição das consequências da perda auditiva, como também promove a independência e bem-estar dos idosos ^40^.

O relatório divulgado pela OMS referente à prevenção de quedas em idosos define esse evento como previsível e evitável ^7^. Evidencia-se a necessidade de ações tendo em mente ambas problemáticas - a perda auditiva e a ocorrência de quedas - visto que são relacionadas, considerando os fatores extrínsecos (o ambiente), intrínsecos (o idoso), comportamentais e a capacitação assistencial dos servidores públicos ^41^.

A chegada da pandemia de COVID-19 gerou uma gama de desafios para a saúde pública, e suas ramificações extrapolaram as próprias infecções pelo vírus. Um dos segmentos mais impactados por essa situação foi a população idosa, principalmente os mais vulneráveis ^42^. O principal achado desta pesquisa pode ter sido “intensificado” pelas ramificações do isolamento social; no entanto, é importante ressaltar a necessidade desse isolamento. Durante a pandemia, pesquisadores identificaram que houve uma elevada incidência de fragilização entre os idosos de idade avançada ^43^, possivelmente decorrente do acesso limitado a cuidados de saúde e à reabilitação (incluindo auditiva), índices menores de atividade física e aumento de estresse e ansiedade.

Destaca-se que algumas limitações devem ser consideradas ao interpretar os resultados deste estudo. Embora o autorrelato da perda auditiva apresente limitações devido à sua natureza subjetiva, estudos indicaram que uma única questão apresenta bom desempenho na identificação de idosos com perda auditiva e pode ser recomendada em pesquisas epidemiológicas, caso não seja possível realizar a avaliação audiológica ^19^ ^,^ ^44^ ^,^ ^45^. Gates et al. ^45^ encontraram uma sensibilidade de 71% e especificidade de 72% para a questão da autopercepção auditiva, enquanto Gates et al. ^44^ encontraram sensibilidade e especificidade de 89,9% e 86,9%, respectivamente. Ainda, pode-se mencionar um possível viés de informação com relação ao autorrelato de quedas nos últimos 12 meses. Outro aspecto está relacionado ao fato de que as análises foram realizadas em uma subamostra dos participantes do estudo EpiFloripa Idoso, os quais concordaram em realizar entrevistas por telefone no período de 2021 a 2022. Os estudos de associação, sobretudo em população idosa, são vulneráveis ao viés de sobrevida, considerando que os idosos mais frágeis e, portanto, com maior frequência tanto de perda auditiva quanto de quedas, podem apresentar maior probabilidade de óbito, não estando representados na amostra. Esse aspecto é uma limitação do nosso estudo que pode diluir a força das associações pesquisadas e subestimar os resultados. Assim, destaca-se que a amostra não é representativa da população idosa do Município de Florianópolis, inviabilizando inferência para a população.

Por fim, constatou-se que, após ajuste do modelo para fatores de confusão, pessoas idosas de uma subamostra do estudo EpiFloripa Idoso que se mantiveram com dificuldade auditiva entre as duas ondas apresentaram 181% mais chance de sofrer queda, quando comparado com seus pares sem dificuldade auditiva. Dessa subamostra, mulheres tiveram maior ocorrência de quedas, sendo a faixa etária de 70 a 79 anos a que registrou maior frequência do evento.

Em última análise, sugere-se que pesquisas futuras aprofundem a compreensão da relação entre a perda auditiva e o risco de quedas, investigando mecanismos subjacentes e explorando abordagens que englobam a intervenção e a prevenção. Por meio da colaboração entre profissionais da saúde e pesquisadores, pode-se propor que a população idosa experiencie um envelhecimento mais saudável e independente.
